# Diagnostic value of qualitative and quantitative parameters of contrast-enhanced ultrasound for differentiating differentiated thyroid carcinomas from benign nodules

**DOI:** 10.3389/fendo.2023.1240615

**Published:** 2024-01-04

**Authors:** Jinfang Fan, Lingling Tao, Weiwei Zhan, Weiwei Li, Lijun Kuang, Yingyan Zhao, Wei Zhou

**Affiliations:** ^1^ Department of Ultrasound, RuiJin Hospital, LuWan Branch, School of Medicine, Shanghai Jiaotong University, Shanghai, China; ^2^ Department of Ultrasound, Ruijin Hospital, School of Medicine, Shanghai Jiao Tong University, Shanghai, China

**Keywords:** thyroid, thyroid carcinoma, contrast-enhanced ultrasound, quantitative, qualitative

## Abstract

**Objective:**

To explore the diagnostic value of contrast-enhanced ultrasound (CEUS) of qualitative and quantitative parameters for differentiating differentiated thyroid cancers from benign nodules.

**Method:**

A total of 290 thyroid nodules that were pathologically confirmed were enrolled in this study. The univariate analysis was performed for the clinical characteristics and CEUS qualitative and quantitative parameters of the inside and peripheral zone of nodules, including age, gender, nodule size, intensity of enhancement, homogeneity, wash-in and wash-out patterns, margin after CEUS, ring enhancement, peak intensity, sharpness, time to peak(TP), and area under the curve(AUC), and the meaningful indicators in the single-factor analysis were further included in multivariate logistic regression analysis.

**Results:**

Multivariate analysis showed that there were significant differences in age (p=0.031), nodule size (p<0.001), heterogeneous enhancement (p<0.001), hypo-enhancement (p=0.001), unclear margin after CEUS(p=0.007), inside peak (p<0.001), and outside sharpness(p<0.001) between benign and malignant nodules. However, there were no significant differences in gender, ring enhancement, wash-in, wash-out, outside TP, outside AUC between benign and malignant thyroid nodules (P>0.05, for all).

**Conclusion:**

CEUS might be useful in the differential diagnosis of differentiated thyroid cancers and benign nodules, which could provide a certain basis for clinical treatment.

## 1 Introduction

Differentiated thyroid cancers (DTC) (1)including papillary thyroid carcinoma (PTC), follicular thyroid carcinoma (FTC), and their variant subtypes (2), are the most frequent endocrine malignancies, and the incidence increased rapidly in recent years. DTC usually has a good prognosis, and Iodine-131 therapy and thyroid inhibitors have been shown to be beneficial for patients 10-year survival rates ranging from 80 to 95 percent (3, 4). However, about 5%-20% of cases may have tumor biological variation due to gene mutation, resulting in different subtypes and poor prognosis, which may be related to the biological characteristics of highly invasive tumors (5). Therefore, the differential diagnosis of thyroid nodules is still of great significance.

Contrast-enhanced ultrasound (CEUS) can evaluate microcirculation perfusion of tissues in real-time (6), providing accurate and reliable data, and it can avoid diagnostic errors caused by individual differences (7). Due to the abundance of micro-vessels in normal thyroid tissue, it shows rapid and homogeneous enhancement after administration of contrast agents. However, thyroid nodules have different angiogenesis patterns, and the manifestations on CEUS may be different (8). Previous studies have reported the CEUS characteristics of thyroid nodules, however, most of them were based on the interior of nodules (9–11), and the enhancement patterns of thyroid nodules on CEUS were still not sufficient to diagnose thyroid cancer (12). So far, there has been only one study focusing on the CEUS characteristics of the peripheral zone of nodules (13). The aim of this study was to evaluate the value of CEUS in the differential diagnosis of DTC by studying the qualitative and quantitative parameters of the internal and peripheral zone of thyroid nodules.

## 2 Materials and methods

### 2.1 Patients

This was a retrospective study, and it was approved by the Ethics Committee of the Ruijin Hospital Luwan Branch, Shanghai Jiao Tong University School of Medicine. A total of 274 patients who underwent surgery for thyroid nodules in our hospital between Mar 2017 and Jul 2021 were included in this study, and 290 nodules were finally analyzed. Inclusion criteria were: (1) Those who signed the informed consent before CEUS examination; (2) The nodules could be completely displayed in one section on US; (3) The final pathological results after surgical excision were obtained. Exclusion criteria were the following: (1) CEUS showed no enhancement; (2) CEUS images were not clear; (3) The distance between the edge of the nodule (>50%) and the thyroid capsule was <3 mm.

### 2.2 US procedure

US examinations were performed with ultrasound instruments (Aplio500, Canon Medical Systems, Tokyo, Japan and Esaote Mylab90, Esaote, Genoa, Italy). Linear array probes (14L5/LA523) with a frequency of 4~13MHz were used for conventional US, and linear array probes (14L5/LA522) with a frequency of 3~9MHz were used for CEUS. The CEUS parameters were set as follows: mechanical index (0.07/0.06), and direct sound pressure (50kPa) (14). The patient was placed in a supine position, fully exposing the neck. The size (maximum diameter) of thyroid nodule was recorded on conventional US, and the section with the most abundant blood flow on power Doppler was selected as the CEUS observation section. The CEUS imaging mode was used, and 1.2ml SonoVue (Bracco, Italy) was then injected through the superficial vein of the elbow, followed by a bolus of 10.0ml normal saline.

### 2.3 Image analysis

All cases were evaluated by two radiologists with more than 3 years of experience in thyroid CEUS. The qualitative CEUS indexes included intensity of enhancement, homogeneity, wash-in, wash-out, margin after CEUS, ring enhancement. Compared to the surrounding thyroid parenchyma, the intensity of enhancement was classified as hyper-enhancement, iso-enhancement, and hypo-enhancement (15). The enhancement homogeneity was divided into homogeneous and heterogeneous, which was based on whether the contrast agent was evenly distributed in lesions. Wash-in and wash-out patterns referred to the enhancement that appeared or disappeared earlier, equal to, or later than the peri-nodular tissue (16). Margin after CEUS was divided into clear and unclear (17). Ring enhancement was defined as an enhanced rim of peritumoral tissue that appeared in the early phase and became more distinct in the late phase, and it was divided into absent and present. Dynamic images were observed for 3min and stored on the hard disk for further analysis.

**Figure 1:**
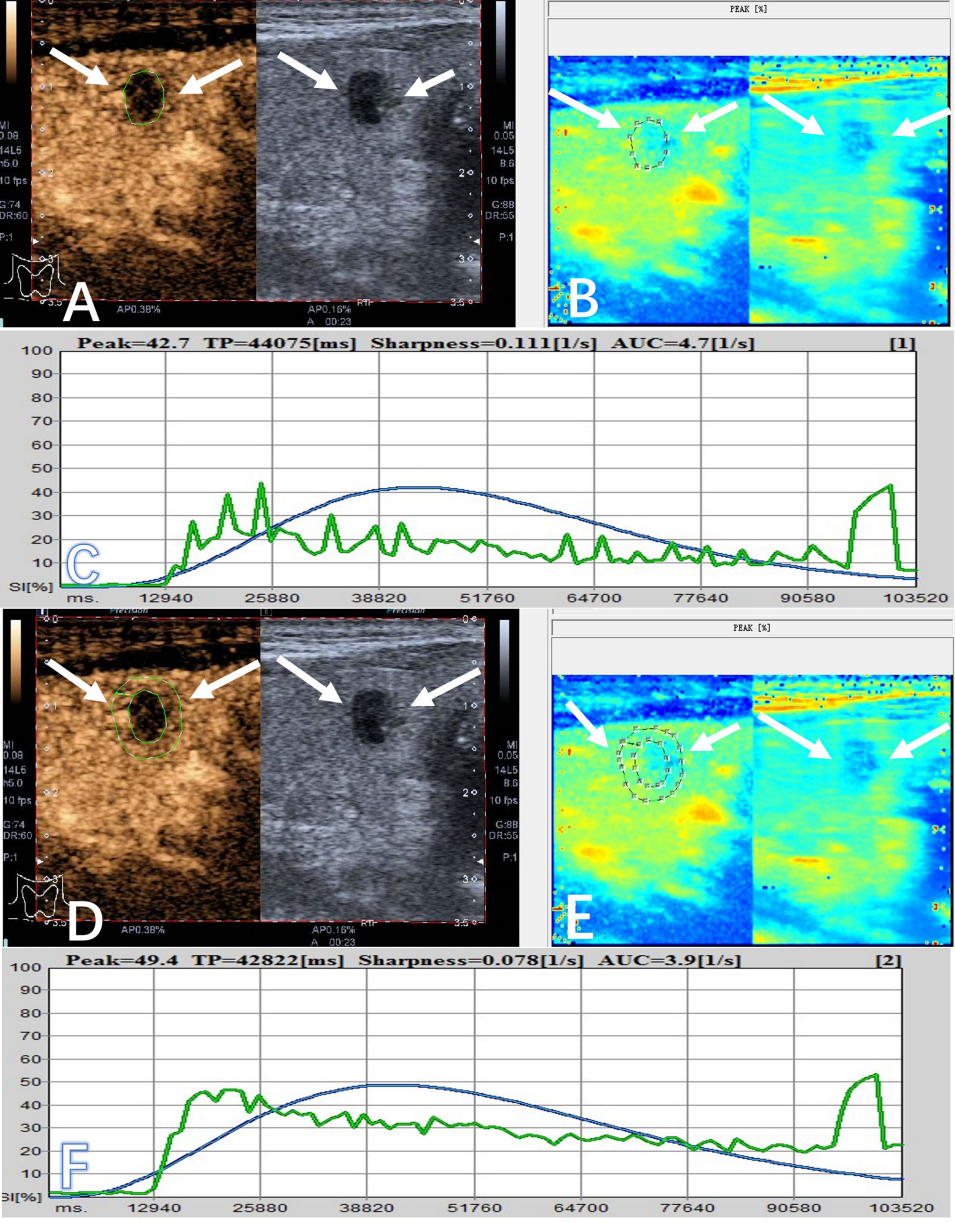
A 56-year-old male patient had a PTC (arrows) in the right lobe, which appeared as a hypoechoic solid nodule with a maximum diameter of 7.0 mm. **(A)** The area of interest was delineated around the interior of nodule on CEUS (arrow), and it showed heterogeneous enhancement and hypo-enhancement on CEUS. **(B)** It showed the peak-mode ROC of the nodule on CEUS. **(C)** The quantitative parameters of the interior of the nodule were obtained from the TIC, including inside peak (42.7%), inside sharpness (0.111 1/s), inside TP (44075 ms), and inside AUC (4.7 1/s). **(D)** The area of interest was selected at the peripheral zone of nodule on CEUS (arrow). **(E)** It showed the peak-mode ROC of the peripheral zone of the nodule on CEUS. **(F)** The quantitative indicators of the peripheral zone were obtained from the TIC, including outside peak (49.4%), outside sharpness (0.078 1/s), outside TP (42822 ms), and outside AUC (3.9 1/s).

The quantitative parameters were analyzed by QontraXt software (Qontraxt Bracco Italy). Two radiologists were blinded to the pathological data. The peak enhancement mode of each target nodule was selected for analysis. The region of interest (ROI) along the maximum outer diameter of the nodule was outlined in order to obtain the time-intensity curve (TIC) of the whole nodule (**Figures 1A–C**), and then the peripheral annular ROI was delineated about 2-3 mm outside the maximum diameter of the nodule to obtain the TIC of the outer edge (**Figures 1D–F**). Each image was analyzed three times, and the average value of the three measurements was taken. The CEUS parameters included: (1) peak (peak intensity, %); (2) sharpness (ascend slope, 1/s); (3) TP (time to peak, ms); (4) AUC (area under the curve, 1/s).

### 2.4 Statistical methods

The statistical analysis was performed by SPSS 21.0 statistical software. Data of quantitative parameters were presented as mean ± standard deviation. Kolmogorov-Smirnov (K-S)test was used to test whether the measurement data conformed to a normal distribution, and independent sample t-test and paired sample t-test were used for measurement data with normal distribution. A non-parametric test was adopted for measurement data with non-normal distribution. Meaningful indicators in the single-factor analysis were further included in logistic regression analysis. The receiver operating characteristic curve (ROC) was used to determine the diagnostic threshold. P value <0.05 was considered to be statistically significant.

## 3 Results

### 3.1 Basic clinical characteristics and pathological results of patients

A total of 274 patients (66 males, 208 females) were enrolled in this study. Patients’ age ranged from 20 to 81 years (mean, 47.9 ± 13.7 years). There were 175 malignant nodules, including 169 PTCs and 6 FTCs. There were 115 benign nodules, including 107 nodular goiters and 8 thyroid adenomas. The size of benign nodules ranged from 3.9 to 61.0mm, with an average of 20.9 ± 13.13 mm, and the size of malignant nodules ranged from 3.1 to 40.2 mm, with an average of 11.6 ± 6.7 mm.

### 3.2 Single factor analysis results of clinical characteristics and CEUS qualitative parameters

According to the literatures (18, 19), age and nodule size were divided in binary manner, and the thresholds were set at 45 years old and 10mm, respectively. The single factor analysis results of clinical characteristics were listed in **Table 1**, and it showed that there were significant differences in gender, age and nodule size between benign and malignant groups (p<0.001, for all). The single factor analysis results of qualitative parameters were listed in **Table 2**, and it indicated that hypo-enhancement (p<0.001), heterogeneous enhancement (p<0.001), unclear margin after CEUS(p<0.001), without ring enhancement (p=0.033), wash-in later (p<0.001), and wash-out earlier (p<0.001) were more commonly detected in malignant nodules than in benign nodules.

**Table 1:** Single factor analysis results of clinical data of thyroid nodules.

parameter	Benign (115)	malignant (175)	p	X^2^
Gender			0.01	6.897
Male	17 (14.8%)	49 (28%)		
Female	98 (85.2%)	126 (72%)		
Age			<0.001	17.859
<45(y)	31 (27.0%)	91 (52.0%)		
≥45(y)	84 (73.0%)	84 (48.0%)		
Nodule size			<0.001	45.588
≤10(mm)	33 (28.7%)	121 (69.1%)		
>10(mm)	82 (71.3%)	54 (30.9%)		

**Table 2:** Single factor analysis results of CEUS qualitative parameters of thyroid nodules.

Parameter	benign (115)	malignant (175)	p	X^2^
Intensity Enhancement			<0.001	55.126
Hyper-enhancement	40 (34.8%)	19 (10.9%)		
Hypo-enhancement	25 (21.7%)	116 (66.3%)		
Iso-enhancement	50 (43.5%)	40 (22.8%)		
homogeneity			<0.001	38.839
Homogeneous	71 (61.7%)	44 (25.1%)		
Heterogeneous	44 (38.3%)	131 (74.9%)		
Ring enhancement			0.033	4.916
Absent	97 (84.3%)	162 (92.6%)		
Present	18 (15.7%)	13 (7.4%)		
Wash-in			<0.001	21.529
Earlier	35 (30.4%)	50 (28.6%)		
Later	31 (27.0%)	94 (53.7%)		
Equal	49 (42.6%)	31 (17.7%)		
Wash-out			<0.001	34.848
Earlier	23 (20.0%)	96 (54.9%)		
Later	13 (11.3%)	21 (12.0%)		
Equal	79 (68.7%)	58 (33.1%)		
Margin after CEUS			<0.001	55.416
Clear	108 (93.9%)	92 (52.6%)		
Unclear	7 (6.1%)	83 (47.4%)		

### 3.3 Single factor analysis results of CEUS quantitative parameters

The results of CEUS quantitative parameters between benign and malignant thyroid nodules were listed in **Table 3**. There were significant differences in inside peak, outside TP, outside sharpness and outside AUC. The inside peak, outside sharpness and outside AUC of malignant nodules were significantly lower than those of benign nodules (p<0.001, for all). The outside TP of malignant nodules was significantly higher than that of benign nodules (p=0.001). **Table 4** showed the diagnostic efficacy of these CEUS quantitative parameters. The sensitivity, specificity, accuracy, and AUC were listed in **Table 4**, respectively.

**Table 3:** Comparison of the inside and outside CEUS quantitative parameters of benign and malignant thyroid nodules.

Pathological type	Peak (%)		TP (ms)		Sharpness (1/s)		AUC (1/s)	
	Outside	Inside	Outside	Inside	Outside	Inside	Outside	Inside
Malignant (175)	31.4 ± 9.9	25.3 ± 10.8	48879 ± 18166	43760 ± 16575	0.138 ± 0.060	0.150 ± 0.082	4.1 ± 1.4	3.9 ± 1.9
Benign (115)	32.2 ± 9.1	33.7 ± 8.2	42300 ± 14138	45392 ± 18620	0.181 ± 0.050	0.139 ± 0.056	4.7 ± 1.3	4.0 ± 1.6
t	0.701	7.146	-3.285	0.781	6.431	-1.343	3.682	0.555
p	0.484	<0.001	0.001	0.436	<0.001	0.180	<0.001	0.579

Peak, peak intensity; TP, time to peak; Sharpness, ascend slope; AUC, area under the curve; Data were presented as mean ± standard deviation.

**Table 4:** Diagnostic efficacy of CEUS quantitative parameters.

Parameter	AUC (%)	p	Benign (115)	Malignant (175)	Sensitivity (%)	Specificity (%)	Accuracy (%)
Inside peak	80.5	<0.001			70.4	80.6	76.6
>29.8			81 (70.4%)	34 (19.4%)			
≤29.8			34 (29.6%)	141 (80.6%)			
Outside TP	61.2	0.001			77.1	41.7	63.1
>36965			67 (58.3%)	135 (77.1%)			
≤36965			48 (41.7%)	40 (22.9%)			
Outside Sharpness	78.3	<0.001					
>0.159			84 (73.0%) 31 (27.0%)	45 (25.7%) 130 (74.3%)	73.0	74.3	73.8
≤0.159							
Outside AUC	63.5	<0.001			84.3	38.9	56.9
≥3.6			97 (84.3%)	107 (61.1%)			
<3.6			18 (15.7%)	68 (38.9%)			

Peak, peak intensity; TP, time to peak; Sharpness, ascend slope; AUC, area under the curve.

### 3.4 Multifactor analysis results of clinical characteristics and CEUS qualitative and quantitative parameters of thyroid nodules

The multifactor analysis results of clinical characteristics and CEUS qualitative and quantitative parameters of thyroid nodules were listed in **Table 5**. It showed that there were significant differences in age (p=0.031), nodule size (p<0.001), heterogeneous enhancement (p<0.001), hypo-enhancement (p=0.001), unclear margin after CEUS (p=0.007), inside peak (p<0.001), and outside sharpness (p<0.001) between benign and malignant nodules.

**Table 5:** Multifactor analysis of clinical and CEUS qualitative and quantitative parameters of thyroid nodules.

Parameter	B	S.E,	Wals	p	Exp(B)	EXP(B) 95% C. I
Gender	0.103	0.564	0.033	0.855	1.108	0.367~3.350
Age	-1.036	0.481	4.629	0.031	0.355	0.138~0.912
Nodule size	-3.214	0.810	15.734	<0.001	0.040	0.008~0.197
Ring enhancement	-0.426	0.759	0.315	0.574	0.653	0.147~2.892
Margin after CEUS	1.706	0.636	7.192	0.007	5.508	1.583~19.166
homogeneity	3.672	0.820	20.072	<0.001	39.327	7.889~196.034
Intensity enhancement	1.676	0.516	10.543	0.001	5.342	1.943~14.686
Wash-in	0.517	0.557	0.861	0.354	1.677	0.563~4.998
Wash-out	1.513	0.781	3.754	0.053	4.540	0.983~20.976
Inside Peak	2.179	0.499	19.102	<0.001	8.835	3.326~23.472
Outside TP	0.570	0.527	1.171	0.279	1.768	0.630~4.966
Outside Sharpness	2.230	0.509	19.175	<0.001	9.298	3.427~25.225
Outside AUC	0.356	0.575	0.383	0.536	1.428	0.463~4.406
Constant	-3.330	1.017	10.718	0.001	0.036	

Peak, peak intensity; TP, time to peak; Sharpness, ascend slope; AUC, area under the curve.

## 4 Discussion

Gray scale ultrasound imaging has been widely used to identify thyroid nodules, however, it is still difficult to differentiate atypical benign and malignant nodules, and previous studies showed that the diagnostic sensitivity was only 27–63% (20). In order to stratify the risk of malignancy in thyroid nodules, the Thyroid Imaging Reporting and Data System (TIRADS) has been established and applied in clinical practice worldwide. However, there is still an overlap of cancer risk between benign and malignant nodules in category 4 or 5 (21). Ultrasound elastography is a non-invasive diagnostic method that can measure tissue stiffness. Li et al. reported that the combination of ultrasound elastography and TIRADS might improve the diagnostic efficacy in category 4 nodules (22). Currently, FNAB has been regarded as the reference standard for the diagnosis of thyroid nodules. The recommendation for FNAB is based on a nodule’s TIRADS level and maximum dimension. However, it still remains controversial to determine the size threshold of suspicious nodules for FNAB in different management guidelines (23, 24). Moreover, there is a challenging point that is being able to make decision as far as suitable management of the nodules with controversial cytology results, including atypia of undetermined significance and follicular lesion of undetermined significance (25). Although molecular testing is recommended for indeterminate cytology by the 2015 ATA Management Guidelines, it may not reliably rule out malignancy with a negative result in this population (24). Hence, there is a need for a definitive evaluation in the clinical examination for thyroid nodules. Zhu et al. reported that the combination of ultrasound elastography with FNAB may improve the diagnostic performance in the differential diagnosis of thyroid nodules (26). According to the European Federation of Societies for Ultrasound in Medicine and Biology guidelines, CEUS was a promising non-invasive method for differentiating benign and malignant thyroid nodules. The type of tumors can be determined by analyzing the enhancement pattern, and the indicators including homogeneity and ring enhancement may provide useful information in the differential diagnosis of thyroid nodules (27). However, the interpretation of CEUS was highly dependent on physicians’ experience and somewhat subjective, and the data of CEUS qualitative parameters overlapped in benign and malignant nodules.

The blood perfusion status of thyroid nodules was related to the number, structure and distribution of blood vessels, and the kinetics of microbubble contrast agent in different thyroid nodules was different. The quantitative analysis technique of CEUS can be used for the quantitative evaluation of blood perfusion in solid organs, and it has also been applied in thyroid nodules. The quantitative CEUS parameters were associated with malignancies and histological type of thyroid nodules (28, 29). The blood vessels may be different in benign and malignant tumors with different pathological classifications and pathological processes, as well as the different regions of the same type of tumor (30). Because of the high interstitial pressure in the tumor, more blood vessels were compressed and collapsed. Therefore, the blood supply in the tumor might be reduced (31). Recently, some studies have focused attention on the area around the tumor. Previous researchers have studied the surrounding zone of breast and liver tumors and found that it might provide new characteristic auxiliary information, which could help surgeons make correct decisions (32, 33). This study investigated the inside and outside CEUS parameters of thyroid nodules in order to improve the diagnostic accuracy of CEUS.

Previous studies suggested (9, 34–36) that hypo-enhancement was closely related to malignant tumors, which was consistent with our data. In this study, hypo-enhancement was more commonly observed in malignant nodules (66.3%) than in benign nodules (21.7%), and the peak intensity of malignant nodules was significantly lower than that of benign nodules. Bartolotta et al (37)reported that the enhancement patterns of thyroid nodules were related to tumor size, and it showed no significant enhancement in malignant thyroid nodules <1 cm. In our study, the proportion of nodule size≤ 10mm was higher in malignant group. Small tumors did not form a large number of mature tumor vascular beds, resulting in insufficient blood supply, so no obvious enhancement could be seen (38–40). Moreover, PTC often showed dense interstitial fibrosis (41). The increase of blood vessels was usually related to cell proliferation in the tumor state, and fibrosis might reduce the vascular density in the nodules (41). Therefore, malignant thyroid nodules were more likely to present hypo-enhancement on CEUS.

According to the Guidelines for the Diagnosis and Treatment of Adult Thyroid Nodules and Differentiated Thyroid Cancer issued by the American Thyroid Association (ATA) in 2015, unclear edge on conventional US was an important indicator for the diagnosis of malignant nodules (24, 42). Yi et al (17) reported that unclear margin after CEUS was also an independent risk factor for malignant thyroid tumors. In this study, the proportion of unclear margins after CEUS in malignant nodules (47.4%) was also significantly higher than that of benign nodules (6.1%). This might be related to the invasiveness of malignant tumor. The blood vessels in the peripheral zone of malignant tumors were relatively dense, which could lead to the tumor invading outwards easily. CEUS focused on the microvascular pattern of tumor. The unclear margins on CEUS might be more likely to reflect the aggressiveness of tumor.

Zhang et al. (14) reported that heterogeneous enhancement was helpful for detecting malignant lesions, and it had a diagnostic sensitivity, specificity and accuracy of 88.2%, 92.5%, and 90.4%, respectively. Xi et al (15) also found that uneven enhancement was more common in malignant thyroid tumors. In this study, the vast majority (74.9%) of malignant nodules showed heterogeneous enhancement. This may be due to the different distribution of new blood vessels in tumor. The neovascularization of malignant lesions was divided into peripheral and central area. The blood vessels were relatively sparse in the central area, and incomplete or complete necrosis occurred easily. Moreover, microthrombus may be present in malignant tumors, which can lead to vascular stenosis or occlusion. Moreover, most vessels in malignant tumor were in a state of low efficacy, which meant that not all of the tumor vessels were in the open state and functional status. Therefore, the neovascularization in the whole lesion was uneven and complex, which might lead to uneven enhancement (39).

The ascend slope of thyroid nodule on CEUS directly reflected the wash-in phase characteristics, and it was recognized as an index for differentiating thyroid nodules. Zhou et al. (11) reported that a low ascend slope was more commonly detected in malignant nodules. However, Petrasova et al. (36) found that there was no statistically significant difference in wash-in slope concerning the biological nature of thyroid nodules. Although the mean value of sharpness in malignant nodules was higher than that in benign nodules in our study, no significant difference was observed (p=0.180). To the best of our knowledge, there were few studies about the ascend slope of the peripheral zone of thyroid nodules. Our results showed that peri-nodular sharpness of malignant nodules was significantly lower than that of benign nodules (p<0.001), which indicated that the enhancement speed of peripheral zone in malignant nodules was slower. Nodular goiter was regarded as a nodular and hyperplastic lesion, which was formed by focal proliferation. The vessel distribution around the tumor was regular with few vessels destroyed by the tumor, so there was no significant difference in the vessel distribution between benign nodules and the peripheral thyroid tissue. The biological behavior of malignant nodules was characterized by invasive growth, destruction of surrounding thyroid tissues and its normal blood supply, and obstruction or interruption of blood flow may occur at the outer edge (39).Therefore, a low ascend slope was more common in malignant nodules.

In this study, it showed that the proportion of malignant nodules (69.1%) ≤10mm was significantly higher than that of benign nodules (28.7%), which was consistent with the results reported by Zheng et al. (43). Lei et al. (44) reported that there was a significant difference in age between benign and malignant thyroid nodules, which was consistent with our data. According to the literature, we set the age threshold at 45 years old (18). In our study, patients younger than 45 years old accounted for more than 52.0% of DTC cases and only 27.0% of benign cases. The reason may be due to the diversification of detection methods, the high sensitivity of detection instruments, and the improvement of people’s health awareness, which could make some small DTCs detected earlier (45, 46).

The present study has some limitations. First, this was a retrospective, single-center study with a relatively small sample size. Second, in the analysis of CEUS, the outline of ROI was not completely consistent in different radiologists. Although the average value was taken, the deviation still existed, and a more scientific method should be performed in the future. Finally, the comparison of physiological specimens after thyroidectomy with CEUS was not performed, which could potentially provide clues in the search for the early warning indicators of aggressive thyroid cancers.

## 5 Conclusion

The qualitative and quantitative analysis of CEUS might be useful in the differential diagnosis of differentiated thyroid cancers and benign nodules, which could provide a certain basis for clinical treatment.

## Data availability statement

The raw data supporting the conclusions of this article will be made available by the authors, without undue reservation.

## Ethics statement

The studies involving humans were approved by the Ethics Committee of the Ruijin Hospital Luwan Branch, Shanghai Jiao Tong University School of Medicine. The studies were conducted in accordance with the local legislation and institutional requirements. The participants provided their written informed consent to participate in this study.

## Author contributions

Study concept and design: JF, WZ, and LT. Data acquisition: JF, WWZ, and LT. Technical and material support: JF, WL, and LT. Data analysis: JF, LK, and LT. Drafting and revision of the manuscript: JF, YZ, and LT. All authors contributed to the article and approved the submitted version.
